# Elaiophylin Elicits Robust Anti-Tumor Responses via Apoptosis Induction and Attenuation of Proliferation, Migration, Invasion, and Angiogenesis in Pancreatic Cancer Cells

**DOI:** 10.3390/molecules28207205

**Published:** 2023-10-21

**Authors:** Lufen Huang, Yufeng Liu, Yiru Pan, Chao Liu, Huijie Gao, Qiang Ren, Jianan Wang, Huiyun Wang, Yuntao Zhang, Anguo Wu

**Affiliations:** 1 Department of Pharmacy, Jining Medical University, Rizhao 276500, China; huanglufen0029@126.com (L.H.); lyf19790408@163.com (Y.L.); lcanatomy@163.com (C.L.); mianyigao@163.com (H.G.); renqiang@mail.jnmc.edu.cn (Q.R.); anansen@163.com (J.W.); wang_huiyun@126.com (H.W.); 2 Sichuan Key Medical Laboratory of New Drug Discovery and Drugability Evaluation, Key Laboratory of Medical Electrophysiology of Ministry of Education, School of Pharmacy, Southwest Medical University, Luzhou 646000, China; pyru2497@163.com

**Keywords:** pancreatic cancer, elaiophylin, BxPC-3, PANC-1, HUVECs, Wnt/β-Catenin

## Abstract

Pancreatic cancer remains a formidable challenge in oncology due to its aggressive nature and limited treatment options. In this study, we investigate the potential therapeutic efficacy of elaiophylin, a novel compound, in targeting BxPC-3 and PANC-1 pancreatic cancer cells. We comprehensively explore elaiophylin’s impact on apoptosis induction, proliferation inhibition, migration suppression, invasion attenuation, and angiogenesis inhibition, key processes contributing to cancer progression and metastasis. The results demonstrate that elaiophylin exerts potent pro-apoptotic effects, inducing a substantial increase in apoptotic cells. Additionally, elaiophylin significantly inhibits proliferation, migration, and invasion of BxPC-3 and PANC-1 cells. Furthermore, elaiophylin exhibits remarkable anti-angiogenic activity, effectively disrupting tube formation in HUVECs. Moreover, elaiophylin significantly inhibits the Wnt/β-Catenin signaling pathway. Our findings collectively demonstrate the multifaceted potential of elaiophylin as a promising therapeutic agent against pancreatic cancer via inhibition of the Wnt/β-Catenin signaling pathway. By targeting diverse cellular processes crucial for cancer progression, elaiophylin emerges as a prospective candidate for future targeted therapies. Further investigation of the in vivo efficacy of elaiophylin is warranted, potentially paving the way for novel and effective treatment approaches in pancreatic cancer management.

## 1. Introduction

Pancreatic cancer is one of the most aggressive and devastating malignancies, with a persistently dismal prognosis and limited treatment options [1]. It ranks as the seventh leading cause of cancer-related deaths worldwide, and its incidence continues to rise [2]. Despite substantial efforts in cancer research, the five-year survival rate for pancreatic cancer remains extremely low, at approximately 10%, underscoring the urgent need for novel therapeutic strategies [3]. Pancreatic cancer is characterized by its rapid progression [4], late-stage diagnosis [4], and resistance to conventional therapies [5]. The majority of patients present with advanced disease, precluding curative surgical intervention and rendering systemic therapies less effective [6]. Additionally, pancreatic tumors are surrounded by a dense stromal microenvironment, which contributes to treatment resistance and immune evasion [7]. Current treatment modalities for pancreatic cancer primarily include surgery [8], chemotherapy [9], radiation therapy [10], and targeted therapies [11]. However, surgery is often limited to a minority of patients, and chemotherapy regimens offer only modest survival benefits [8]. Moreover, acquired resistance to chemotherapy agents further complicates treatment outcomes [9].

Elaiophylin, a novel small molecule compound, has gained significant attention for its purported anti-cancer effects [12,13,14]. Preclinical studies have suggested that elaiophylin possesses the ability to trigger apoptosis and inhibit proliferation in various cancer cell lines [12,13,14]. However, its efficacy and potential mechanisms of action in pancreatic cancer remain largely unexplored. Therefore, in this study, we investigate the therapeutic potential of elaiophylin in targeting BxPC-3 pancreatic cancer cells, seeking to shed light on its comprehensive anti-cancer properties.

Apoptosis, or programmed cell death, plays a fundamental role in maintaining tissue homeostasis by eliminating damaged or abnormal cells [15]. Dysregulation of apoptosis is a hallmark of cancer, allowing cancer cells to evade cell death and promote uncontrolled proliferation [16]. We hypothesize that elaiophylin may act as a potent pro-apoptotic agent, inducing programmed cell death in BxPC-3 cells, and thereby impeding tumor growth. Moreover, uncontrolled cell proliferation is a fundamental feature of cancer. Elaiophylin’s potential to inhibit cancer cell proliferation is of particular interest, as it may halt the unbridled growth of pancreatic cancer cells, offering an additional therapeutic avenue. Furthermore, the metastatic spread of cancer cells is a critical determinant of patient prognosis. Cell migration and invasion are key processes that facilitate cancer cell dissemination to distant sites [17]. We postulate that elaiophylin may possess anti-migratory and anti-invasive properties, potentially hampering the metastatic cascade in BxPC-3 cells. Angiogenesis, the formation of new blood vessels from pre-existing vasculature, is crucial for tumor growth and metastasis, providing essential nutrients and oxygen to the rapidly proliferating cancer cells [18]. Inhibition of angiogenesis has been recognized as an effective strategy to curtail tumor progression. Here, we aim to explore the impact of elaiophylin on angiogenesis, seeking to elucidate its potential as an anti-angiogenic agent in pancreatic cancer. In summary, this study explores the multifaceted anti-cancer effects of elaiophylin in pancreatic cancer BxPC-3 cells, encompassing apoptosis induction, proliferation inhibition, migration suppression, invasion attenuation, and anti-angiogenesis. We believe that this investigation will contribute valuable insights into the potential utility of elaiophylin as a novel therapeutic agent for combating pancreatic cancer, potentially paving the way for new targeted therapeutic strategies to improve patient outcomes.

## 2. Results

### 2.1. Elaiophylin Inhibits the Proliferation of BxPC-3 and PANC-1 Cells

**Figure 1:**
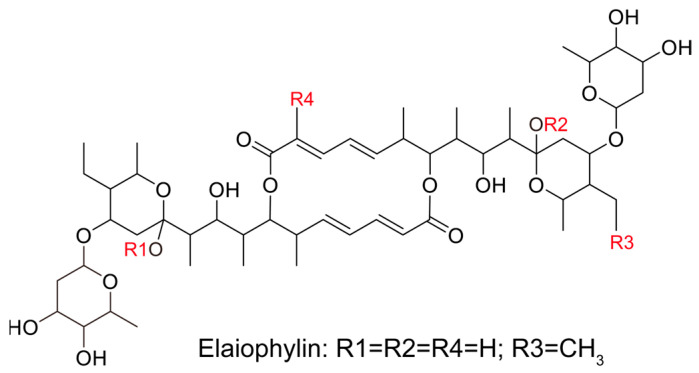
The chemical structure of elaiophylin.

**Figure 2:**
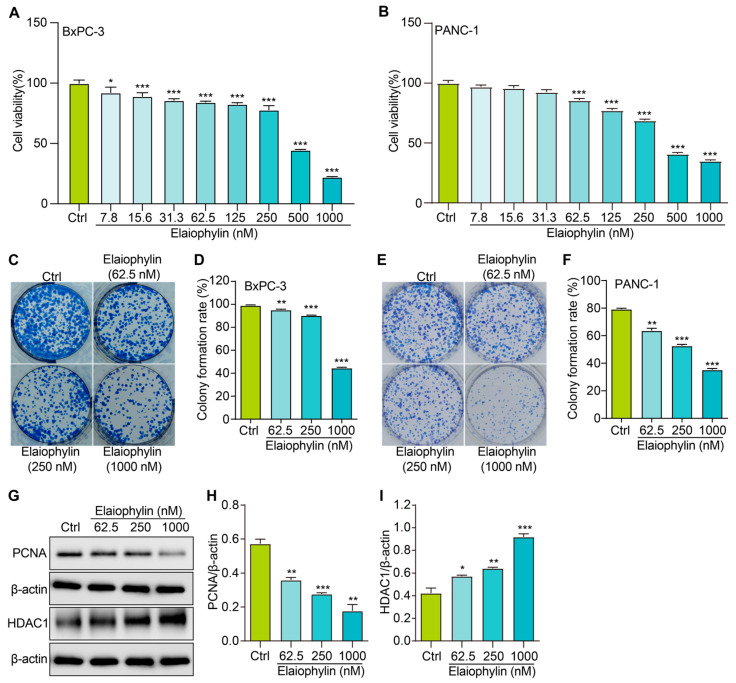
Elaiophylin inhibits the proliferation of BxPC-3 and PANC-1 cells. (**A**) The bar chart indicates the cell viability of BxPC-3 cells treated with elaiophylin at indicated concentrations. (**B**) The bar chart indicates the cell viability of PANC-1 cells treated with elaiophylin at indicated concentrations. (**C**,**E**) Representative images of colony formation of BxPC-3 and PANC-1 cells treated with elaiophylin at indicated concentrations. (**D**,**F**) The bar chart indicates the colony-formation rate. (**G**) Representative western blotting images of PCNA, HDAC1, and β-actin in BxPC-3 cells treated with elaiophylin at indicated concentrations. The original western blotting images are shown in Figures S2 and S3. (**H**,**I**) Bar charts indicate the ratio of PCNA/β-actin and HDAC1/β-actin. Error bars, S.D.: * *p* < 0.05; ** *p* < 0.01; *** *p* < 0.001. Data are presented as mean ± SD from three independent experiments *(n* = 3).

In this study, we evaluated the potential anti-proliferative effect of elaiophylin, an active compound isolated from the mycelium of Streptomyces hygroscopicus LP-93, on BxPC-3 and PANC-1 cells by using a variety of in vitro assays; the chemical structure of elaiophylin is shown in Figure 1. To evaluate the impact of elaiophylin on cell viability and proliferation, we performed the CCK-8 assay. BxPC-3 and PANC-1 cells were treated with various concentrations of elaiophylin (ranging from 7.8 to 1000 nM) for 24 h. The results demonstrated a dose-dependent decrease in cell viability upon elaiophylin treatment, with IC_50_ values for the two cell types of 452.8 nM and 467.7 nM, respectively, indicating potent growth inhibition (Figure 2A,B). Additionally, we have assessed the activity of elaiophylin on the normal cell lines HPDE and HUVEC. The results indicate that these normal cell lines undergo less cell death under the same treatment conditions, with IC_50_ values of 499.4 nM and 498.9 nM for HPDE cells and HUVECs, respectively (Figure S1A,B). To further assess the anti-proliferative potential of elaiophylin, we conducted the colony-formation assay. BxPC-3 and PANC-1 cells were treated with elaiophylin at the indicated concentrations. Control cells were treated with a concentration of DMSO equivalent to which used for the 1000 nM elaiophylin treatment. This ensured that any observed effects were attributable to elaiophylin and not the solvent. The results revealed substantial decreases in the number and size of colonies in the elaiophylin-treated group compared to the control group, indicating an inhibitory effect on cell proliferation (Figure 2C–F). To gain insights into the molecular mechanisms underlying the anti-proliferative action of elaiophylin, we assessed the inhibitory effects of elaiophylin on BxPC-3 cell proliferation by evaluating Proliferating Cell Nuclear Antigen PCNA (PCNA) and Histone Deacetylase 1 (HDAC1) protein expression. Western blot analysis revealed a decrease in PCNA expression and an increase in HDAC1 protein content after elaiophylin treatment (Figure 2G–I). This suggests that elaiophylin may exert its anti-proliferative effects on BxPC-3 cells by downregulating PCNA and upregulating HDAC1 expression. Taken together, our comprehensive results demonstrate that elaiophylin exerts a potent anti-proliferative effect on BxPC-3 and PANC-1 pancreatic cancer cells.

### 2.2. Elaiophylin Promotes the Cell-Cycle Arrest of BxPC-3 Cells

**Figure 3:**
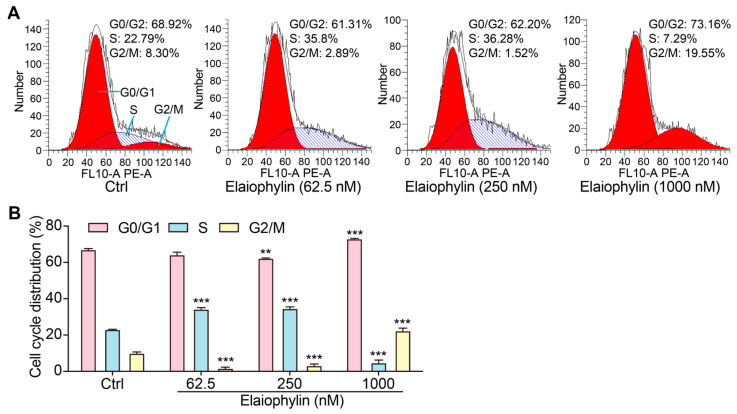
Elaiophylin promotes the cell-cycle arrest of BxPC-3 cells. (**A**) Representative flow cytometry images showing the cell cycle of elaiophylin-treated BxPC-3 cells stained with PI reagent. The peaks in the FACS graph represent cells in the G0/G1, S, and G2/M phases of the cell cycle. The shaded area indicates the cell population with specific DNA content, representing cells in different phases of the cell cycle. (**B**) The bar chart indicates the cell-cycle distribution rate. Error bars, S.D.: ** *p* < 0.01; *** *p* < 0.001. Data are presented as mean ± SD from three independent experiments (*n* = 3).

To investigate the potential impact of elaiophylin on the cell-cycle distribution of BxPC-3 pancreatic cancer cells, we employed the PI single staining flow cytometry method. After treatment, cells were collected, fixed, and stained with PI reagent to label cellular DNA. The stained cells were then subjected to flow cytometry analysis. Flow cytometry analysis of the PI-stained BxPC-3 cells, as shown in Figure 3A,B, indicated significant alterations in the cell-cycle distribution upon elaiophylin treatment. Compared to the control group, elaiophylin-treated cells exhibited a notable increase in the percentage of cells arrested at the G0/G1 phase (72.94% ± 0.32% in 1000 nM elaiophylin-treated vs. 66.97% ± 0.83% in control). Concurrently, there was a marked decrease in the proportion of cells in the S phase (4.73% ± 1.87% in 1000 nM elaiophylin-treated vs. 23.04% ± 0.16% in control) and G2/M phase (1.62% ± 0.80% and 3.19% ± 1.05% in 62.5 and 250 nM elaiophylin-treated vs. 15.7% ± 1.0% in control). Our results demonstrate that elaiophylin at concentrations of 62.5 and 250 nM predominantly induces cell-cycle arrest at the S phase, whereas 1000 nM elaiophylin leads to cell-cycle arrest at both the G0/G1 and G2/M phases in BxPC-3 pancreatic cancer cells.

### 2.3. Elaiophylin Induces Cell Apoptosis in BxPC-3 Cells

**Figure 4:**
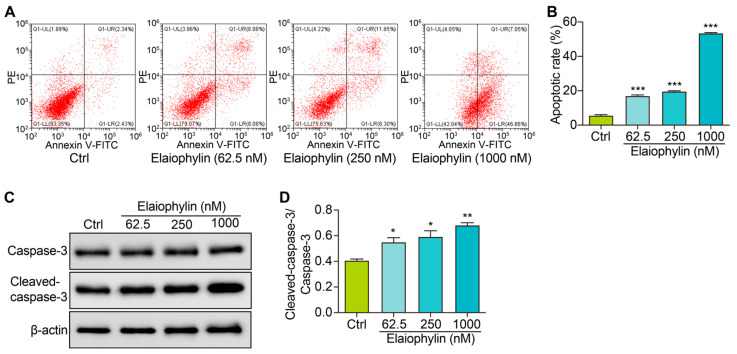
Elaiophylin induces cell apoptosis in BxPC-3 cells. (**A**) Representative flow cytometry images showing the cell apoptosis of elaiophylin-treated BxPC-3 cells stained with Annexin V-FITC and PI reagent. (**B**) The bar charts indicate the cell apoptotic rate. (**C**) Representative western blotting images of Caspase-3, Cleaved-caspase-3, and β-actin in BxPC-3 cells treated with elaiophylin at indicated concentrations. The original western blotting images are shown in Figure S4. (**D**) The bar charts indicate the ratio of Cleaved-caspase-3/caspase-3. Error bars, S.D.: * *p* < 0.05; ** *p* < 0.01; *** *p* < 0.001. Data are presented as mean ± SD from three independent experiments *(n* = 3).

To investigate the potential pro-apoptotic effect of elaiophylin on BxPC-3 pancreatic cancer cells, we determined the cell apoptotic rate and the protein expression of cleaved-caspase-3. After treatment, the cells were harvested and stained with Annexin V-FITC and PI according to the manufacturer’s instructions. Flow cytometry analysis was then performed to evaluate the percentage of cells undergoing apoptosis. Flow cytometry results revealed a significant increase in the percentage of apoptotic cells following elaiophylin treatment. Compared to the control group, elaiophylin-treated cells exhibited a dose-dependent elevation in early apoptotic cells (Annexin V-FITC positive, PI negative) and late apoptotic cells (Annexin V-FITC positive, PI positive) (Figure 4A,B). This indicates that elaiophylin treatment induced both early and late stages of apoptosis in BxPC-3 cells. To further confirm the pro-apoptotic effect of elaiophylin, we performed western blot analysis to assess the protein expression of Cleaved-caspase-3, a key executioner caspase in the apoptotic pathway. Figure 4C,D show that elaiophylin treatment led to a significant increase in the protein expression of Cleaved-caspase-3 compared to the control group. The elevated levels of Cleaved-caspase-3 protein further support the idea of induction of apoptosis by elaiophylin in BxPC-3 cells. Our results demonstrate that elaiophylin exerts a pro-apoptotic effect on BxPC-3 pancreatic cancer cells.

### 2.4. Elaiophylin Inhibits the Migration and Invasion of BxPC-3 and PANC-1 Cells

**Figure 5:**
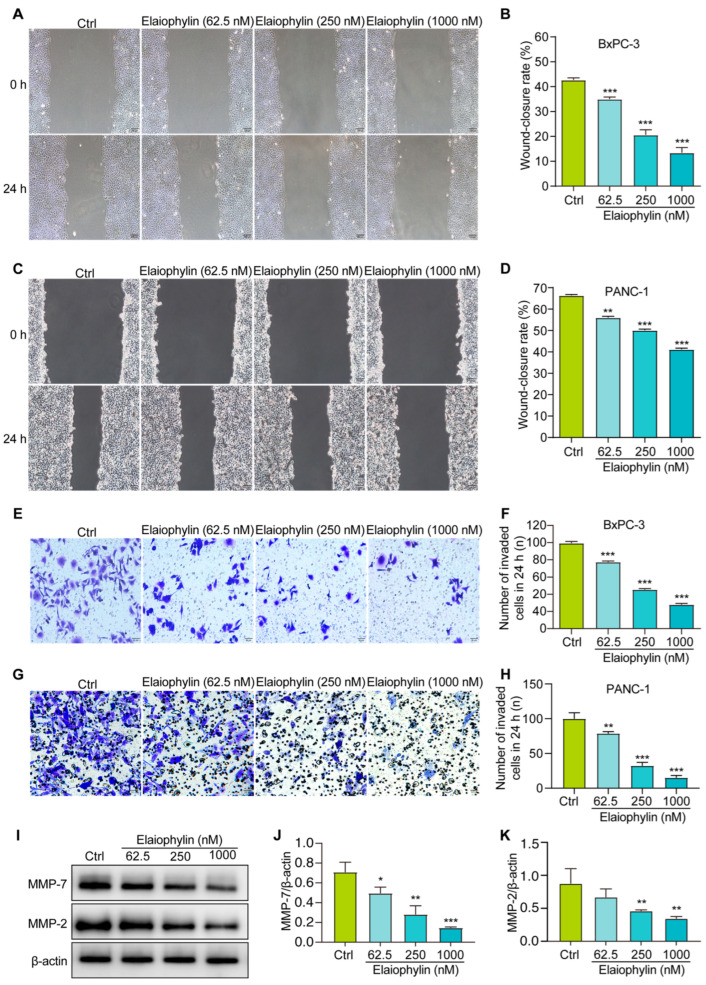
Elaiophylin inhibits the migration and invasion of BxPC-3 and PANC-1 cells. (**A**,**C**) Representative cell scratch images of BxPC-3 and PANC-1 cells treated with elaiophylin at indicated concentrations. Magnification: 10×, scale bar: 100 µm. (**B**,**D**) The bar chart indicates the wound-closure rates of BxPC-3 and PANC-1 cells. (**E**,**G**) Representative cell invasive images of elaiophylin-treated BxPC-3 and PANC-1 cells stained with crystal violet reagent. Magnification: 20×, scale bar: 50 µm. (**F**,**H**) The bar chart indicates the number of invaded cells in 24 h. (**I**) Representative western blotting images of MMP-7, MMP-2, and β-actin in BxPC-3 cells treated with elaiophylin at indicated concentrations. The original western blotting images are shown in Figure S5. (**J**,**K**) Bar charts indicate the ratio of MMP-7/β-actin and MMP-2/β-actin. Error bars, S.D.: * *p* < 0.05; ** *p* < 0.01; *** *p* < 0.001. Data are presented as mean ± SD from three independent experiments (*n* = 3).

To investigate the potential anti-migratory effect of elaiophylin on BxPC-3 and PANC-1 pancreatic cancer cells, the scratch assay was employed. Results from the scratch assay revealed that elaiophylin treatment significantly inhibited cell migration in a dose-dependent manner. Compared to the control group, elaiophylin-treated cells exhibited reduced migration rates, indicating the potential of elaiophylin to impede the migratory capacity of BxPC-3 and PANC-1 cells (Figure 5A,B). To further investigate the effect of elaiophylin on cell invasion, we conducted the Transwell assay. BxPC-3 and PANC-1 cells were treated with elaiophylin at various concentrations and then seeded into the upper chamber of the Transwell inserts, which were coated with Matrigel. The ability of cells to invade through the Matrigel and migrate to the lower chamber was evaluated. The Transwell assay results demonstrated that elaiophylin treatment led to a significant decrease in cell invasion compared to the control group. The reduced number of invaded cells in the lower chamber indicated the anti-invasive potential of elaiophylin in BxPC-3 and PANC-1 cells (Figure 5E–H). To gain insights into the molecular mechanisms underlying elaiophylin’s impact on migration and invasion, we performed western blot analysis to assess the protein expression of MMP-7 and MMP-2, two key matrix metalloproteinases associated with cancer cell migration and invasion. Figure 5I–K show that elaiophylin treatment resulted in a significant downregulation of MMP-7 and MMP-2 protein expression in BxPC-3 cells compared to the control group, suggesting elaiophylin’s potential in inhibiting the expression of enzymes/proteases involved in cancer cell migration and invasion. Our results indicate that elaiophylin exhibits potent anti-migratory and anti-invasive effects in BxPC-3 pancreatic cancer cells.

### 2.5. Elaiophylin Inhibits Angiogenesis

**Figure 6:**
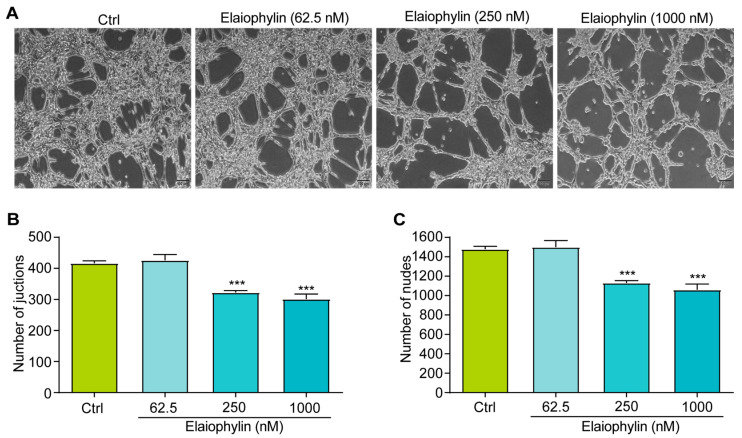
Elaiophylin inhibits angiogenesis. (**A**) Representative images of elaiophylin-treated HUVECs showing the formation of capillary-like structures. Magnification: 10×, scale bar: 100 µm. (**B**) The bar chart indicates the number of junctions. (**C**) The bar chart indicates the number of nodes. Error bars, S.D.: *** *p* < 0.001. Data are presented as mean ± SD from three independent experiments (*n* = 3).

The tube formation assay is a widely accepted in vitro method to evaluate the ability of endothelial cells to form capillary-like structures, mimicking the process of angiogenesis [19]. In this study, HUVECs were utilized to investigate the potential anti-angiogenic effect of elaiophylin by assessing angiogenesis-related parameters, including the number of junctions and the number of nodes. Junctions represent the points where three or more endothelial cells converge to form a branching point, while nodes are defined as the endpoints of tubes. The numbers of both junctions and nodes are crucial parameters reflecting the extent of angiogenic activity [20]. Figure 6A–C revealed a significant reduction in the number of junctions and nodes in HUVECs treated with elaiophylin, compared to the control group. This indicates that elaiophylin treatment effectively inhibited the formation of capillary-like structures, suggesting its potential to suppress angiogenesis.

### 2.6. Elaiophylin Inhibits the Activation of Wnt/β-Catenin Signaling Pathway in BxPC-3 Cells

**Figure 7:**
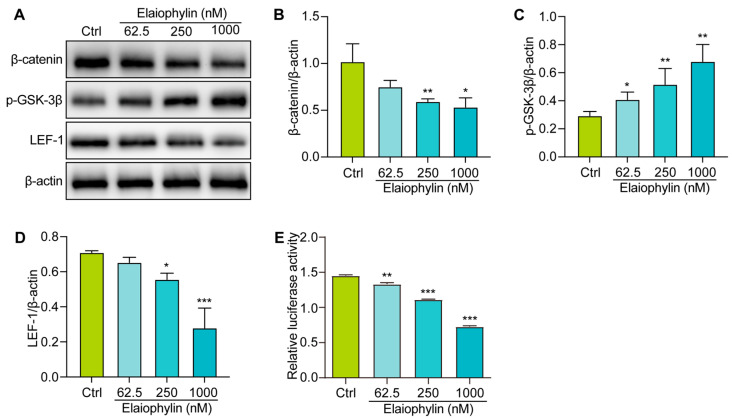
Elaiophylin inhibits the activation of the Wnt/β-Catenin signaling pathway in BxPC-3 cells. (**A**) Representative western blotting images of β-catenin, p-GSK-3β, LEF-1, and β-actin in BxPC-3 cells treated with elaiophylin at indicated concentrations. (**B**–**D**) Bar charts indicate the ratio of β-catenin/β-actin, p-GSK-3β/β-actin, and LEF-1/β-actin. The original western blotting images are shown in Figure S6. (**E**) The bar chart indicates the relative luciferase activity of the TOPFlash firefly luciferase. Error bars, S.D.: * *p* < 0.05; ** *p* < 0.01; *** *p* < 0.001. Data are presented as mean ± SD from three independent experiments (*n* = 3).

To explore the molecular mechanism involved the anti-tumor effect of elaiophylin in BxPC-3 cells, we examined the regulation of elaiophylin on the Wnt/β-Catenin signaling pathway. Western blot analysis revealed that elaiophylin treatment led to a significant downregulation of β-catenin protein expression in BxPC-3 cells, compared to the control group. Additionally, the levels of phosphorylated-GSK-3β (p-GSK-3β), the inactive form of GSK-3β, were increased upon elaiophylin treatment (Figure 7A–D). Furthermore, the protein expression of LEF-1, a downstream transcription factor in the Wnt/β-Catenin signaling pathway, was also reduced following elaiophylin treatment. To further corroborate the inhibitory effect of elaiophylin on Wnt/β-Catenin signaling, we performed the luciferase assay. BxPC-3 cells were transfected with a luciferase reporter plasmid containing TCF/LEF binding sites, along with a control reporter plasmid. The cells were then treated with elaiophylin or the control vehicle. Luciferase activity was measured, and the results showed a significant decrease in the level of β-catenin-mediated TCF/LEF transcription activity in elaiophylin-treated cells compared to the control group (Figure 7E). The results from our study indicate that elaiophylin effectively inhibits the activation of Wnt/β-Catenin signaling in BxPC-3 pancreatic cancer cells.

## 3. Discussion

Pancreatic cancer remains one of the most challenging malignancies in oncology, characterized by its aggressive behavior and limited treatment options [21]. The urgent need for novel therapeutic agents that target multiple facets of this cancer is evident. In this regard, our study offers a comprehensive evaluation of the potential therapeutic effects of elaiophylin, a macrolide antibiotic derived from Streptomyces melanosporus, in pancreatic cancer treatment. Emerging evidence from various cancer cell lines suggests that elaiophylin exerts anti-tumor effects through mechanisms such as inhibiting autophagy/mitophagy [22], triggering paraptosis [13], inducing oxidative stress [23], suppressing angiogenesis [24], and activating endoplasmic reticulum (ER) stress [25]. Key pathways implicated include the inhibition of SIRT1/Nrf2 signaling, MAPK hyperactivation, downregulation of VEGF, and inhibition of SIRT1/FoxO3a signaling. Furthermore, compared to the six analogues of elaiophylin evaluated, elaiophylin itself showcased the most potent anti-tumor effects. Structurally, four pivotal chemical groups (R1–R4) delineate the anti-tumor potency of these analogues, as depicted in Figure 1. In our study, we observed that elaiophylin exerts multiple anti-cancer activities on BxPC-3 pancreatic cancer cells. These include inhibiting cell proliferation, inducing cell-cycle arrest, promoting cell apoptosis, suppressing cell migration and invasion, and inhibiting angiogenesis. While these findings are promising, we recognize that the specific mechanisms of action of elaiophylin in pancreatic cancer warrant further investigation. In future studies, we are committed to delving deeper into these mechanisms to provide a more comprehensive understanding of elaiophylin’s therapeutic potential in pancreatic cancer. This will be instrumental in positioning elaiophylin as a promising candidate for the development of novel therapeutic agents against this formidable cancer.

The first significant finding of this study is the potent anti-proliferative effect of elaiophylin on BxPC-3 cells and PANC-1 cells. The CCK-8 assay showed a dose-dependent decrease in cell viability upon elaiophylin treatment, with IC_50_ values of 452.8 nM and 467.7 nM, respectively. This indicates that elaiophylin significantly inhibits the growth and proliferation of BxPC-3 cells and PANC-1 cells. Furthermore, the safety profile of a therapeutic agent is paramount for its potential clinical application. In this regard, our experiments with normal cells, i.e., HPDE and HUVEC, were reassuring. We observed that elaiophylin imparts almost identical effects to the pancreatic cancer cells and the normal control cell lines across the tested dose–response spectrum. This observation underscores the need to approach the therapeutic potential of elaiophylin with caution. While our results robustly demonstrate the anti-tumor responses in BxPC-3 cells, the overlapping effects on normal cells suggest that the therapeutic window for elaiophylin could be narrow. Further investigations are imperative to delineate the differential molecular mechanisms activated in cancerous versus normal cells in response to elaiophylin. This observation also emphasizes the necessity for careful dosage considerations when contemplating elaiophylin as a therapeutic agent for pancreatic cancer, given its potential effects on normal cells at therapeutic concentrations [13,22,23,24]. In addition, the colony-formation assay further supported the idea that elaiophylin-treated cells formed fewer and smaller colonies compared to the control group. Additionally, the western blot examination showcased a downtrend in PCNA articulation, while concurrently indicating an uptrend in HDAC1 protein accumulation subsequent to elaiophylin intervention. This suggests its regulatory role in cell proliferation. These findings collectively demonstrate that elaiophylin effectively suppresses the proliferation of BxPC-3 cells by interfering with key regulators of cell growth and division. Another important finding of this study is the ability of elaiophylin to induce cell-cycle arrest in BxPC-3 cells. Flow cytometry analysis revealed a notable increase in cells arrested at the S phase when treated with elaiophylin at concentrations of 62.5 and 250 nM. In contrast, 1000 nM elaiophylin resulted in prominent cell-cycle arrest at both the G0/G1 and G2/M phases in BxPC-3 pancreatic cancer cells. This observation underscores the multifaceted nature of elaiophylin’s impact on cell-cycle dynamics. The differential effects observed at varying concentrations suggest that elaiophylin may interact with distinct molecular targets or pathways depending on its concentration. Specifically, while lower concentrations predominantly induce cell-cycle arrest at the S phase, higher concentrations appear to exert a broader inhibitory effect, arresting cells at both the G0/G1 and G2/M phases. This nuanced response to elaiophylin underscores the complexity of its mechanism of action and highlights the need for a deeper understanding of its interactions within the cellular environment. It is plausible that elaiophylin, at different concentrations, modulates the activity or stability of specific cell-cycle regulators, thereby dictating the phase at which cells are arrested. Future studies delving into the molecular underpinnings of these observations are warranted. Such investigations could provide insights into the regulation of the cell cycle by elaiophylin and potentially unveil novel therapeutic targets for pancreatic cancer treatment.

Furthermore, our study demonstrates the pro-apoptotic effect of elaiophylin on BxPC-3 cells. Flow cytometry analysis revealed a significant increase in the percentage of apoptotic cells following elaiophylin treatment, both in early and late stages of apoptosis. Western blot analysis confirmed the elevated protein expression of Cleaved-caspase-3, indicating the activation of the apoptotic pathway. This suggests that elaiophylin induces apoptosis in BxPC-3 cells through the activation of caspases. Apoptosis is a fundamental process for the elimination of damaged or abnormal cells, and its induction is a critical aspect of cancer therapy [26]. Elaiophylin’s ability to induce apoptosis in pancreatic cancer cells makes it an attractive candidate for potential therapeutic interventions. Our study also highlights the anti-migratory and anti-invasive properties of elaiophylin in BxPC-3 and PANC-1 cells. The scratch assay revealed that elaiophylin treatment significantly reduced cell migration, and the Transwell assay demonstrated a decrease in cell invasion. These observations suggest that elaiophylin impairs the migratory and invasive capacity of BxPC-3 and PANC-1 cells, which are essential processes for cancer metastasis. Additionally, western blot analysis revealed a downregulation of MMP-7 and MMP-2 protein expression after elaiophylin treatment, supporting the idea of its potential role in inhibiting the enzymatic activity involved in cancer cell migration and invasion. This indicates that elaiophylin may exert its anti-metastatic effects by modulating key regulators of cell motility and invasiveness. Moreover, our study provides evidence of the anti-angiogenic activity of elaiophylin. The tube formation assay demonstrated a significant reduction in the number of junctions and nodes in HUVECs treated with elaiophylin, indicating the latter’s ability to inhibit the formation of capillary-like structures, a key process in angiogenesis. Targeting angiogenesis is an effective approach for cancer therapy, as it hinders the growth and spread of tumors by cutting off their blood supply. The anti-angiogenic potential of elaiophylin suggests a possible role in suppressing tumor angiogenesis and highlights its broader effects on the tumor microenvironment. The Wnt/β-Catenin signaling pathway plays a pivotal role in various cellular processes, including cell proliferation, differentiation, and apoptosis [27,28,29,30]. Dysregulation of this pathway is frequently observed in cancer and has been associated with tumor initiation, progression, and metastasis [31]. In pancreatic cancer, aberrant activation of the Wnt/β-Catenin pathway has been linked to increased cell proliferation and survival, as well as enhanced epithelial–mesenchymal transition (EMT) and cancer stem cell properties, all of which contribute to the aggressive nature of this disease [32,33]. Our findings suggest that elaiophylin may act as a potent inhibitor of Wnt/β-Catenin signaling, leading to the suppression of cancer-cell growth and survival. By reducing the levels of β-catenin and enhancing the phosphorylation of GSK-3β, elaiophylin likely interferes with the stabilization and nuclear translocation of β-catenin, thereby inhibiting its transcriptional activity. The downregulation of LEF-1, a key transcription factor downstream of β-catenin, further supports the inhibition of Wnt/β-Catenin signaling and its downstream gene expression. Importantly, targeting the Wnt/β-Catenin pathway has emerged as a promising therapeutic strategy for pancreatic cancer treatment. Inhibitors of this pathway have shown potential to reverse the aggressive phenotype of pancreatic cancer cells and sensitize them to conventional chemotherapy. Therefore, elaiophylin’s ability to inhibit the Wnt/β-Catenin pathway adds to its value as a potential therapeutic agent for pancreatic cancer.

In addition to the observed effects in pancreatic cancer BxPC-3 cells, elaiophylin has demonstrated promising anti-tumor properties in various other cell lines and entities. For instance, the anti-tumor efficacy of elaiophylin was demonstrated in ovarian cancers, such as SKOV3, OVCAR8, UWB1.289, and SW626 cells, where a treatment regimen of 0.5 µM was employed [12,13]. Similarly, elaiophylin exhibits potent anti-tumor effects in lung cancers, such as A549 and H1975, and Calu-3 cells, using concentrations of 0.25 and 0.5 µM. Additionally, 0.5-2 µM elaiophylin is reported to effectively inhibit the growth of UM C918 and OCM1A cells [23]. Therefore, these studies, along with our findings, underscore the broad-spectrum anti-tumor potential of elaiophylin.

Given the robust anti-tumor responses elicited by elaiophylin in our study, it is pertinent to consider its potential clinical applications. PDAC patients, especially those with tumors exhibiting resistance to conventional therapies or those with aggressive tumor phenotypes, might benefit from elaiophylin therapy. Our findings suggest that elaiophylin’s ability to induce apoptosis and attenuate proliferation, migration, invasion, and angiogenesis could offer therapeutic advantages in managing aggressive and resistant PDAC tumors. Regarding the concentrations used in our study, which demonstrated significant anti-tumor effects in BxPC-3 cells, it is crucial to note that in vitro concentrations might not directly translate to in vivo dosages due to factors like drug metabolism, distribution, and clearance. However, the concentrations chosen were based on preliminary dose–response studies and are in line with concentrations reported in other studies on elaiophylin’s anti-tumor effects [12,13,23]. Future pharmacokinetic and pharmacodynamic studies are essential to determine the clinically relevant doses of elaiophylin and to assess its safety and efficacy in PDAC patients.

## 4. Materials and Methods

### 4.1. Chemical, Reagent, and Antibodies

Elaiophylin was isolated from the mycelium of Streptomyces hygroscopicus LP-93, with a purity of 99% (HPLC). It was prepared as a stock solution in DMSO and stored at −20 °C. Roswell Park Memorial Institute (RPMI) 1640, penicillin, streptomycin, and fetal bovine serum (FBS) were purchased from Thermo Fisher Scientific (Waltham, MA, USA). Endothelial cell growth medium (ECM) medium was purchased from Sciencell (Carlsbad, CA, USA). The Cell Counting Kit-8 (CCK-8) assay kit was obtained from Dojindo Laboratories (Kumamoto, Japan). Matrigel, an extracellular matrix gel derived from Engelbreth-Holm–Swarm tumor cells, was purchased from BD Biosciences (Franklin Lakes, NJ, USA). This study utilized the following primary antibodies: PCNA (ab29), HDAC1 (ab280198), Pro-caspase-3 (ab32150), Cleaved-caspase-3 (ab214430), β-catenin (ab305261), p-GSK-3β (ab75814), LEF-1 (ab137872), MMP-7 (ab302893), MMP-2 (ab86607), and β-actin (ab8227). These antibodies were obtained from Ningmeng Biotech Co. (Jinan, China), a representative of Abcam (Cambridge, MA, USA). The secondary antibodies, goat anti-mouse and goat anti-rabbit, were purchased from KeyGEN Biotech Co. (Nanjing, China). Transwell plates were purchased from Corning Life Sciences (New York, NY, USA). Crystal violet solution was purchased from Sigma-Aldrich Co. (St. Louis, MO, USA). The Dual Glo-Luciferase assay reagent was purchased from Promega (Madison, WI, USA). Annexin V-FITC/PI Apoptosis Detection Kit and Cell Cycle Kit were purchased from Keygen Biotech Co. (Nanjing, China).

### 4.2. Cell Culture

Pancreatic cancer cell line BxPC-3 cells (Cellosaurus ID: CVCL_0186) without the KRAS mutation and PANC-1 cells (Cellosaurus ID: CVCL_0480) with the KRAS mutation were obtained. Additionally, normal cell lines, including HPDE cells (Cellosaurus ID: CVCL_4376) and human umbilical vein endothelial cells (HUVECs, Cellosaurus ID: CVCL_2959), were sourced. All cell lines were procured from the American Type Culture Collection (ATCC, Manassas, VA, USA) and were subsequently authenticated by Jiangsu KeyGEN BioTECH Corp., Ltd. on 10 September 2021. The BxPC-3, PANC-1, and HPDE cells were cultured in RPMI-1640 medium, whereas the HUVECs were maintained in ECM medium. Both media were supplemented with 10% fetal bovine serum (FBS) and 1% penicillin-streptomycin. Cells were maintained in a humidified incubator at 37 °C with 5% CO_2_.

### 4.3. CCK-8 Assay

The Cell Counting Kit-8 (CCK-8) assay was employed to evaluate cell viability following elaiophylin treatment. Briefly, BxPC-3 and PANC-1 cells were separately incubated with both elaiophylin and DMSO control for 24 h. At the end of the treatment period, CCK-8 solution was added to the culture medium in each well and incubated for an additional 2 h. The formazan dye formed by viable cells was quantified by measuring the absorbance at 450 nm using a microplate reader (BioTek ELx800, Winooski, VT, USA). The experiment was performed in triplicate, and the results were expressed as a percentage of viable cells relative to the control.

### 4.4. Clonogenic Assay

To evaluate the impact of elaiophylin on colony formation, a clonogenic assay was performed. Briefly, after serum starvation, BxPC-3 and PANC-1cells were treated with elaiophylin at different concentrations and seeded into 6-well plates at low cell densities (100–500 cells per well). The plates were then incubated at 37 °C with 5% CO_2_ for 14 days to allow the formation of colonies. During this period, the medium was replenished every 3 days with fresh medium containing the respective elaiophylin concentrations. After the incubation period, cells were fixed with 4% paraformaldehyde and stained with crystal violet. Visible colonies were manually counted, and only colonies containing 50 or more cells were considered for quantification. Data represents the average of three independent experiments performed in triplicate.

### 4.5. Apoptosis Detection

To investigate the induction of apoptosis by elaiophylin, treated and untreated BxPC-3 cells were collected at 24 h. Apoptotic cells were detected using an Annexin V-FITC/propidium iodide (PI) apoptosis detection kit, as has been described previously [34]. Cells were stained with Annexin V-FITC and PI according to the manufacturer’s instructions, followed by the analysis on a flow cytometer (Beckman Coulter, Brea, CA, USA). The percentage of apoptotic cells was determined by analyzing Annexin V-FITC-positive and PI-negative cells using CytExpert 2 software (Beckman Coulter, Brea, CA, USA). Data represents the average of three independent experiments performed in triplicate.

### 4.6. Cell-Cycle Assay

To assess the effects of elaiophylin on the cell-cycle distribution of BxPC-3 cells, a PI (propidium iodide) staining method was employed. After serum-starvation, BxPC-3 cells were treated with different concentrations of elaiophylin and incubated for 24 h. At the end of the treatment period, the cells were harvested by trypsinization and washed with cold PBS. The cells were then fixed in 70% ethanol and stored at −20 °C for at least 1 h to ensure proper fixation. Prior to analysis, the fixed cells were washed with PBS to remove residual ethanol. The fixed cells were resuspended in a PI staining solution containing PI (50 μg/mL), RNase A (100 μg/mL), and Triton X-100 (0.1%) in PBS. The cells were incubated in the dark at 37 °C for 30 min to allow PI staining of the cellular DNA. The stained cells were subjected to flow cytometry analysis, and the data were processed using flow cytometry analysis software to determine the percentage of cells in each cell-cycle phase (G0/G1, S, and G2/M). Data represents the average of three independent experiments performed in triplicate.

### 4.7. Cell Scratching Assay

BxPC-3 and PANC-1 pancreatic cancer cells were seeded in 6-well plates and allowed to reach confluency. Prior to the experiment, the cells were serum-starved for 24 h to synchronize their growth. Using a sterile 200 μL pipette tip, a straight scratch was made across the confluent monolayer of BxPC-3 and PANC-1 cells. Care was taken to ensure that the scratches were of consistent width and length. The detached cells and debris were gently removed by washing the cells with serum-free RPMI-1640 medium. The scratched BxPC-3 and PANC-1 cells were then treated with different concentrations of elaiophylin or vehicle control (solvent) and incubated for 24 h. The scratched area was marked, and time-lapse images of the scratch were acquired at 0 h (immediately after scratching) and at 24 h using an inverted microscope (Olympus IX51, Olympus, Tokyo, Japan). The time-lapse images were analyzed using image analysis software to measure the migration of BxPC-3 and PANC-1 cells into the scratch area. The rate of migration and the extent of wound closure were quantified and compared between elaiophylin-treated groups and the vehicle control group. Data represents the average of three independent experiments performed in triplicate.

### 4.8. Transwell Assays

BxPC-3 and PANC-1 pancreatic cancer cells were seeded in 6-well plates and allowed to reach confluency. Upon reaching 70–80% confluency, the cells were serum-starved for 24 h to synchronize their growth before the experiment. To assess the effect of elaiophylin on cell invasion, a Transwell migration assay was performed using 24-well Transwell inserts with an 8.0 μm pore size. The upper chamber of the Transwell insert was coated with Matrigel to mimic the extracellular matrix (ECM). Briefly, BxPC-3 and PANC-1 cells were resuspended in serum-free RPMI-1640 medium containing elaiophylin at different concentrations and seeded into the upper chamber at a predetermined cell density. In the lower chamber, RPMI-1640 medium was used as the chemoattractant, providing a gradient for cell migration towards the lower surface of the insert. The Transwell plate was incubated for 24 h to allow cells to invade through the Matrigel and migrate to the lower surface. After incubation, the non-migrated cells on the upper surface of the Transwell insert were carefully removed using a cotton swab. Migrated cells on the lower surface of the insert were fixed with 4% paraformaldehyde and stained with crystal violet. Images of the stained cells were captured under a light microscope (Olympus IX51, Japan), and the number of invaded cells was quantified by counting cells in five randomly selected fields for each well. Data represents the average of three independent experiments performed in triplicate.

### 4.9. Angiogenesis Assay

For the angiogenesis assay, HUVECs were seeded in 24-well plates at a predetermined cell density. To assess the anti-angiogenic effects of elaiophylin, an in vitro angiogenesis assay, namely the tube formation assay, was conducted. Briefly, after serum-starvation for 6 h, HUVECs were treated with elaiophylin at different concentrations and seeded onto a layer of growth-factor-reduced Matrigel in the 24-well plates. The plate was incubated for 6 to 8 h to allow tube formation by HUVECs. Tube formation was observed under an inverted microscope to monitor the morphological changes and formation of capillary-like structures. Images of the formed tubes were captured at multiple random fields for each well. Tube length, the number of branch points, and the number of complete tubes were quantified using ImageJ analysis software (Version 2.0, NIH). Data represents the average of three independent experiments performed in triplicate.

### 4.10. Western Blotting

Following the elaiophylin treatment, adherent BxPC-3 cells were carefully washed with ice-cold phosphate-buffered saline (PBS) to remove any residual media and contaminants. The cells were then lysed in radioimmunoprecipitation assay (RIPA) buffer supplemented with protease and phosphatase inhibitors. Cell lysates were collected and centrifuged at high speed, 12,000× *g*, for 15 min at 4 °C to remove cellular debris, and the supernatants containing total protein were collected for the determination of protein concentration using the Bradford assay. Equal amounts of protein from each sample were mixed with loading buffer and denatured by heating at 95 °C for 5 min. The denatured protein samples were loaded onto sodium dodecyl sulfate–polyacrylamide gel electrophoresis (SDS-PAGE) gels for electrophoresis. Following SDS-PAGE, the separated proteins were transferred from the gel to a polyvinylidene difluoride (PVDF) membrane. After transfer, the PVDF membrane was blocked with 5% non-fat dry milk in Tris-buffered saline containing 0.1% Tween 20 (TBST) for 1 h at room temperature to prevent non-specific binding. Subsequently, the membrane was incubated overnight at 4 °C with specific primary antibodies targeting proteins of interest. After incubation, the membrane was washed several times with TBST to remove unbound antibodies. The PVDF membrane was further incubated with appropriate horseradish peroxidase (HRP)-conjugated secondary antibodies for 1 h at room temperature. Following extensive washing, protein bands were visualized using enhanced chemiluminescence (ECL) reagents and detected using a chemiluminescence imaging system. Densitometric analysis was performed to quantify the relative band intensities. The blots shown are representative of at least three independent experiments.

### 4.11. Luciferase Assay

For the luciferase assay, BxPC-3 cells were seeded in 96-well plates at an appropriate density and allowed to adhere overnight. Next, the BxPC-3 cells were transfected with a luciferase reporter plasmid containing a promoter region responsive to elaiophylin treatment, driving the expression of the luciferase gene. Lipofectamine 3000 was used as the transfection reagent according to the manufacturer’s protocol. After transfection for 24 h, the cell culture medium was aspirated, and cells were lysed using a luciferase lysis buffer to release intracellular contents, including luciferase. Equal amounts of protein from each lysate were added to separate wells of a white, opaque 96-well microplate. Subsequently, the luciferase assay substrate, luciferin, was added to each well, initiating the enzymatic reaction with luciferase. The luminescence signal generated by the luciferase–luciferin reaction was measured immediately using a luminometer. Three independent experiments were performed.

### 4.12. Statistical Analysis

All experiments were performed independently at least three times, and data were expressed as mean ± standard deviation (SD). Statistical significance between treatment groups and control was determined using one-way analysis of variance (ANOVA) followed by appropriate post-hoc tests. A *p*-value < 0.05 was considered statistically significant. Statistical analyses were performed using the GraphPad Prism 9.0 software (Boston, MA, USA).

## 5. Conclusions

In conclusion, our study provides valuable insights into the potential of elaiophylin as a novel therapeutic agent for pancreatic cancer. The observed anti-cancer effects of elaiophylin, including apoptosis induction, proliferation inhibition, migration suppression, invasion attenuation, and angiogenesis inhibition, were may partly regulated by the Wnt/β-Catenin signaling pathway. Further translational research is warranted to evaluate the efficacy of elaiophylin in animal models and clinical trials, with the ultimate goal of improving patient outcomes and advancing the field of pancreatic cancer therapeutics.

## Data Availability

The data presented in this study are available on request from the corresponding author.

## Supplementary Materials

The following supporting information can be downloaded at: https://www.mdpi.com/article/10.3390/molecules28207205/s1, Figure S1: The effect of elaiophylin on the cell viability of HPDE cells and HUVEC cells. (A) The bar chart indicates the cell viability of HPDE cells treated with elaiophylin at indicated concentrations. (B) The bar chart indicates the cell viability of HUVEC cells treated with elaiophylin at indicated concentrations; Figure S2: The original Western blotting images for Figure 2G; Figure S3: The original Western blotting images for Figure 2G; Figure S4: The original Western blotting images for Figure 4C; Figure S5: The original Western blotting images for Figure 5I; Figure S6: The original Western blotting images for Figure 7A.
