# Supplementary material for: Elaiophylin Elicits Robust Anti-Tumor Responses via Apoptosis Induction and Attenuation of Proliferation, Migration, Invasion, and Angiogenesis in Pancreatic Cancer Cells
Source: Molecules. 2023 Oct 21;28(20):7205. doi: 10.3390/molecules28207205 (PMC10608934; doi:10.3390/molecules28207205)
Supplement: Supplementary file 1 [file molecules-28-07205-s001.zip › molecules-2556532-supplementary.pdf]

Supplementary materials

Elaiophylin Elicits Robust Anti-Tumor Responses via Apoptosis Induction and Attenuation of Proliferation, Migration, Invasion, and Angiogenesis in Pancreatic Cancer BxPC-3 Cells

Lufen Huang[1], Yufeng Liu[1], Yiru Pan[2], Chao Liu[1], Huijie Gao[1], Qiang Ren[1], Jianan Wang[1], Huiyun Wang[1], Yuntao Zhang[1,*], Anguo Wu[2,*]

[1] Department of Pharmacy, Jining Medical University, Rizhao 276500, China; huanglufen0029@126.com (L.H.); lyf19790408@163.com (Y.L.); lcanatomy@163.com (C.L.); mianyigao@163.com (H.G.); renqiang@mail.jnmc.edu.cn (Q.R.); anansen@163.com (J.W.); wang_huiyun@126.com (H.W.)

[2] Sichuan Key Medical Laboratory of New Drug Discovery and Drugability Evaluation, Key Laboratory of Medical Electrophysiology of Ministry of Education, School of Pharmacy, Southwest Medical University, Luzhou 646000, China; pyru2497@163.com

* Correspondence: zhangyt16@126.com (Y.Z.); wuanguo@swmu.edu.cn (A.W.); Tel.: +86-17769617417 (A.W.)

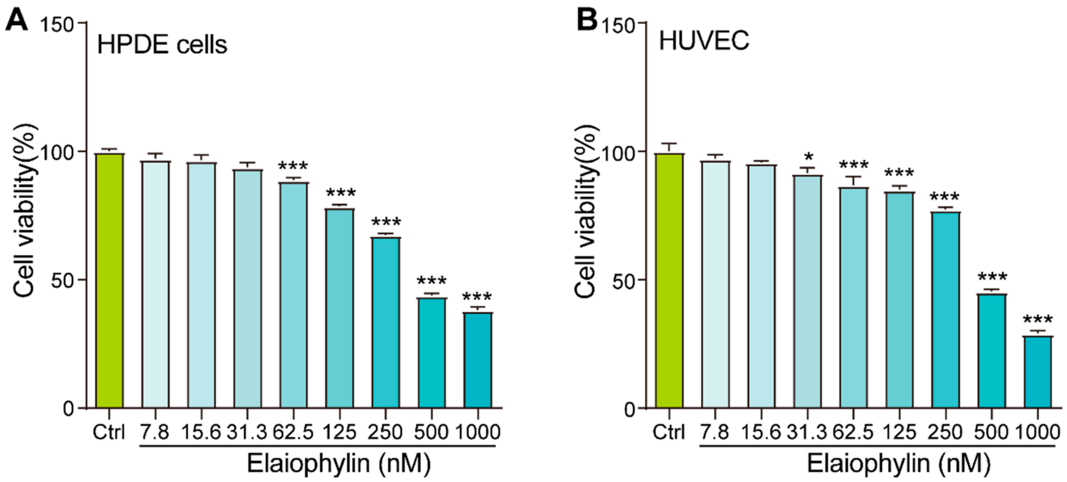

Supplementary Figure S1. The effect of elaiophylin on the cell viability of HPDE cells and HUVEC cells. (A) The bar chart indicates the cell viability of HPDE cells treated with elaiophylin at indicated concentrations. (B) The bar chart indicates the cell viability of HUVEC cells treated with elaiophylin at indicated concentrations. Error bars, S.D., *p < 0.05; ***p < 0.001. Data are presented as mean ± SD from three independent experiments (n=3).

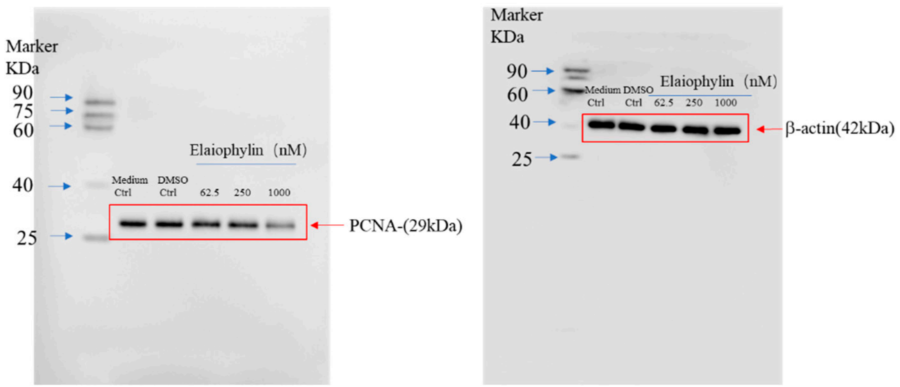

Supplementary Figure S2. The original Western blotting images for Figure 2G

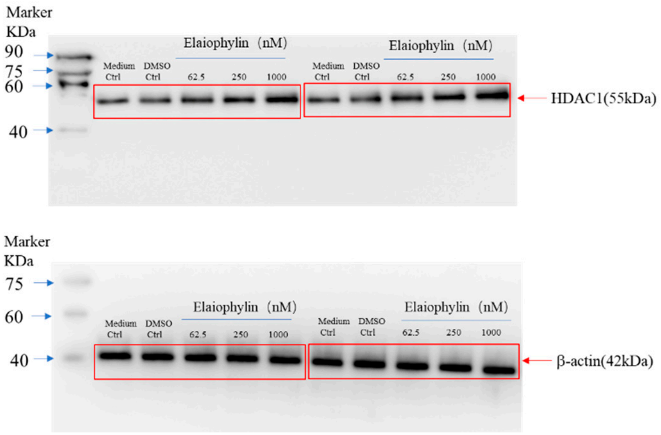

Supplementary Figure S3. The original Western blotting images for Figure 2G

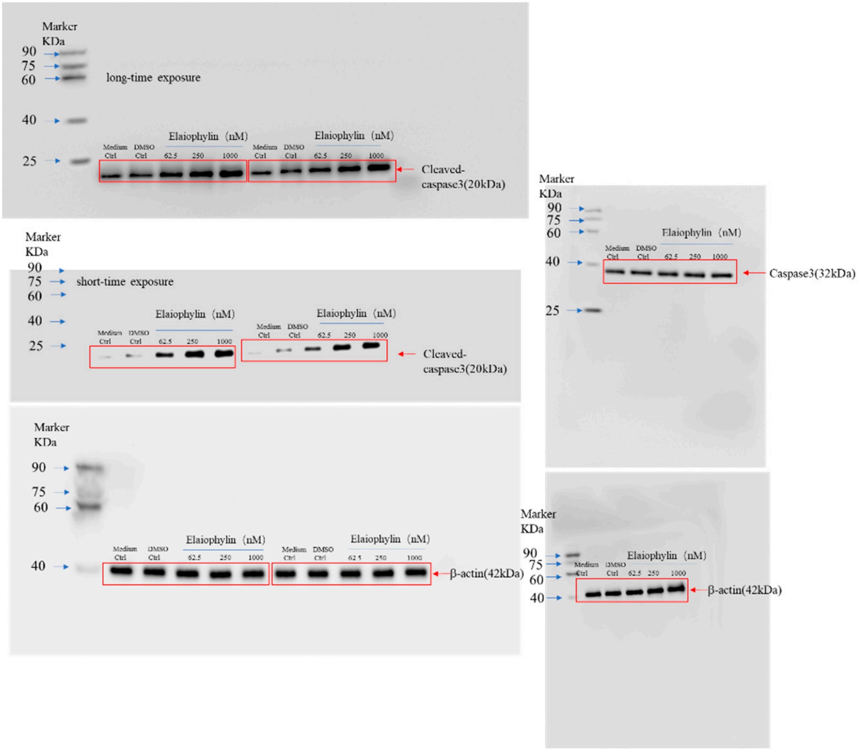

Supplementary Figure S4. The original Western blotting images for Figure 4C

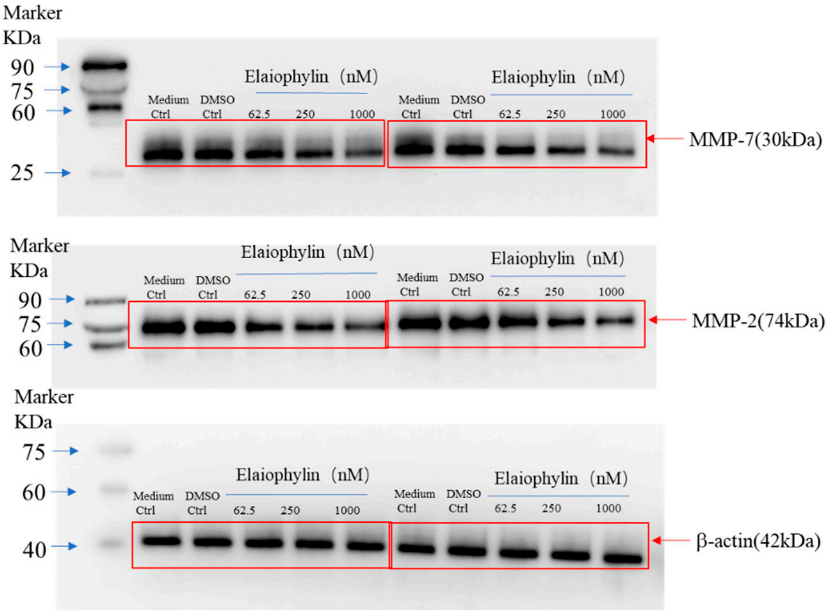

Supplementary Figure S5. The original Western blotting images for Figure 5E

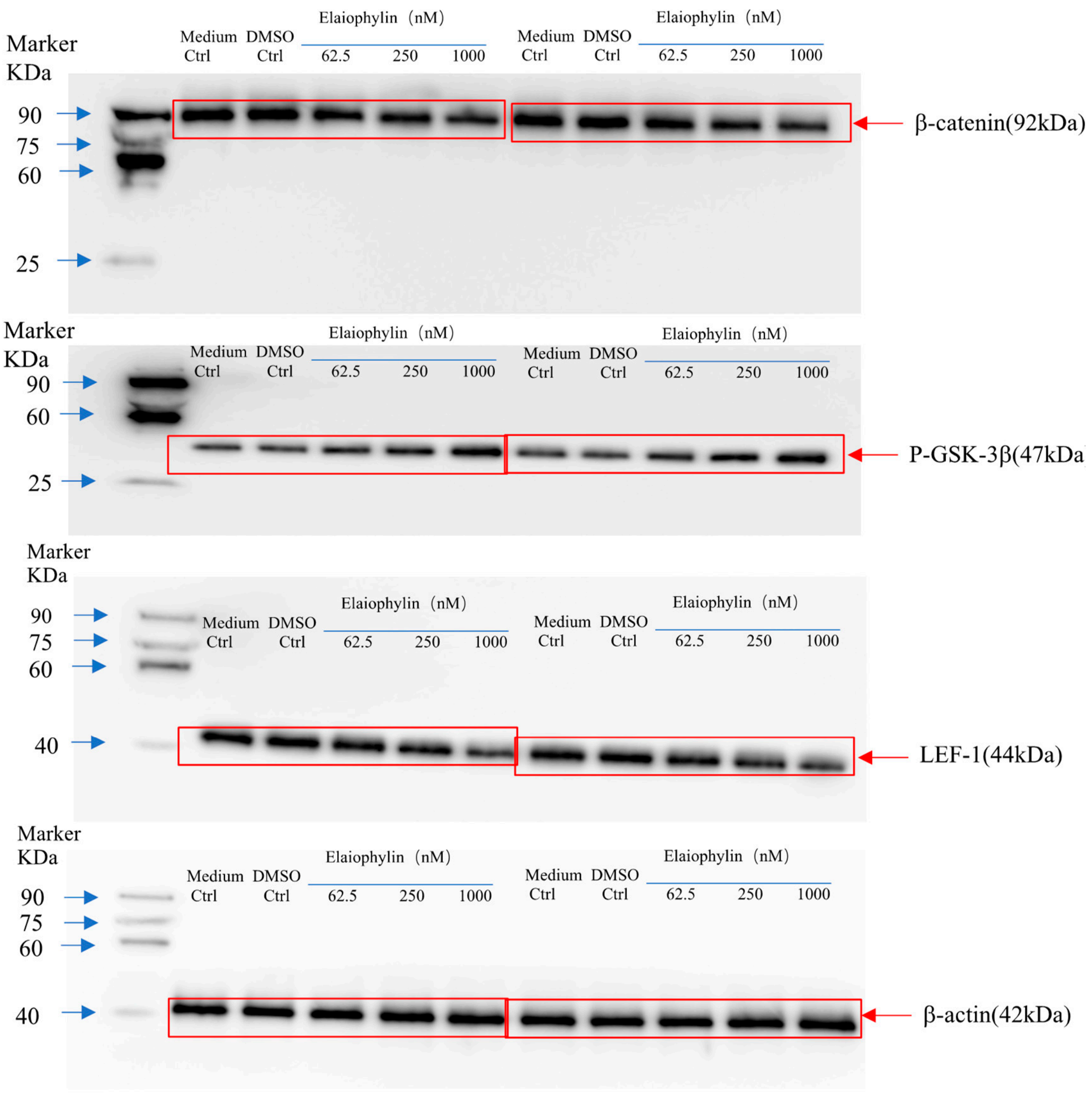

Supplementary Figure S6. The original Western blotting images for Figure 7A
